# Supplementary material for: Genetic Diversity of Aspergillus flavus on Maize in Guatemala
Source: Foods. 2023 Oct 21;12(20):3864. doi: 10.3390/foods12203864 (PMC10606850; doi:10.3390/foods12203864)
Supplement: Supplementary file 1 [file foods-12-03864-s001.zip › New Supplemental files/Supplemental Table S2 ANOVA and Tukey.pdf]

ANOVA for Complete Dataset (272 isolates)

Na

Source	Nparm	DF	Sum of Squares	F Ratio	Prob > F
Pop	3	3	24.54688	8.4251	0.0001*
Locus	15	15	291.23438	19.9918	<.0001*

Level			Least Sq Mean
Med	A		4.8750000
Low	A		4.6250000
Zero	A		4.1875000
High		B	3.2500000

Ne

Source	Nparm	DF	Sum of Squares	F Ratio	Prob > F
Pop	3	3	1.492854	1.4318	0.2460
Locus	15	15	80.197797	15.3833	<.0001*

I

Source	Nparm	DF	Sum of Squares	F Ratio	Prob > F
Pop	3	3	0.125002	1.2317	0.3093
Locus	15	15	19.094946	37.6313	<.0001*

h

Source	Nparm	DF	Sum of Squares	F Ratio	Prob > F
Pop	3	3	0.0139322	0.5885	0.6257
Locus	15	15	4.5008414	38.0245	<.0001*

uh

Source	Nparm	DF	Sum of Squares	F Ratio	Prob > F
Pop	3	3	0.0129105	0.5230	0.6687
Locus	15	15	4.6671664	37.8124	<.0001*

ANOVA for Clones (126 clones)

Na

Source	Nparm	DF	Sum of Squares	F Ratio	Prob > F
Pop	3	3	115.54687	37.5127	<.0001*
Locus	15	15	286.85938	18.6260	<.0001*

Level			Least Sq Mean
Zero	A		5.6875000
Med	A		5.1250000
Low		B	2.8125000
High		B	2.6875000

Ne

Source	Nparm	DF	Sum of Squares	F Ratio	Prob > F
Pop	3	3	5.90032	6.6909	0.0008*
Locus	15	15	143.48701	32.5425	<.0001*

Level			Least Sq Mean
Zero	A		2.7504375
Med	A	B	2.5230625
Low		B	2.0515625
High		B	2.0507500

I

Source	Nparm	DF	Sum of Squares	F Ratio	Prob > F
Pop	3	3	2.550534	35.8519	<.0001*
Locus	15	15	23.536040	66.1675	<.0001*

Level				Least Sq Mean
Zero	A			1.0783125
Med		B		0.8944375
Low			C	0.6121250
High			C	0.6056875

h

Source	Nparm	DF	Sum of Squares	F Ratio	Prob > F
Pop	3	3	0.3619972	23.3194	<.0001*
Locus	15	15	5.2708684	67.9085	<.0001*

Level				Least Sq Mean
Zero	A			0.50500000
Med		B		0.41887500
Low			C	0.32518750
High			C	0.32362500

uh

Source	Nparm	DF	Sum of Squares	F Ratio	Prob > F
Pop	3	3	0.3424110	19.0216	<.0001*
Locus	15	15	5.7205202	63.5571	<.0001*

Level				Least Sq Mean
Zero	A			0.51862500
Med		B		0.42862500
Low			C	0.34256250
High			C	0.34125000
