# Supplementary figures and images for: Genetic Diversity of Aspergillus flavus on Maize in Guatemala
Source: Foods. 2023 Oct 21;12(20):3864. doi: 10.3390/foods12203864 (PMC10606850; doi:10.3390/foods12203864)

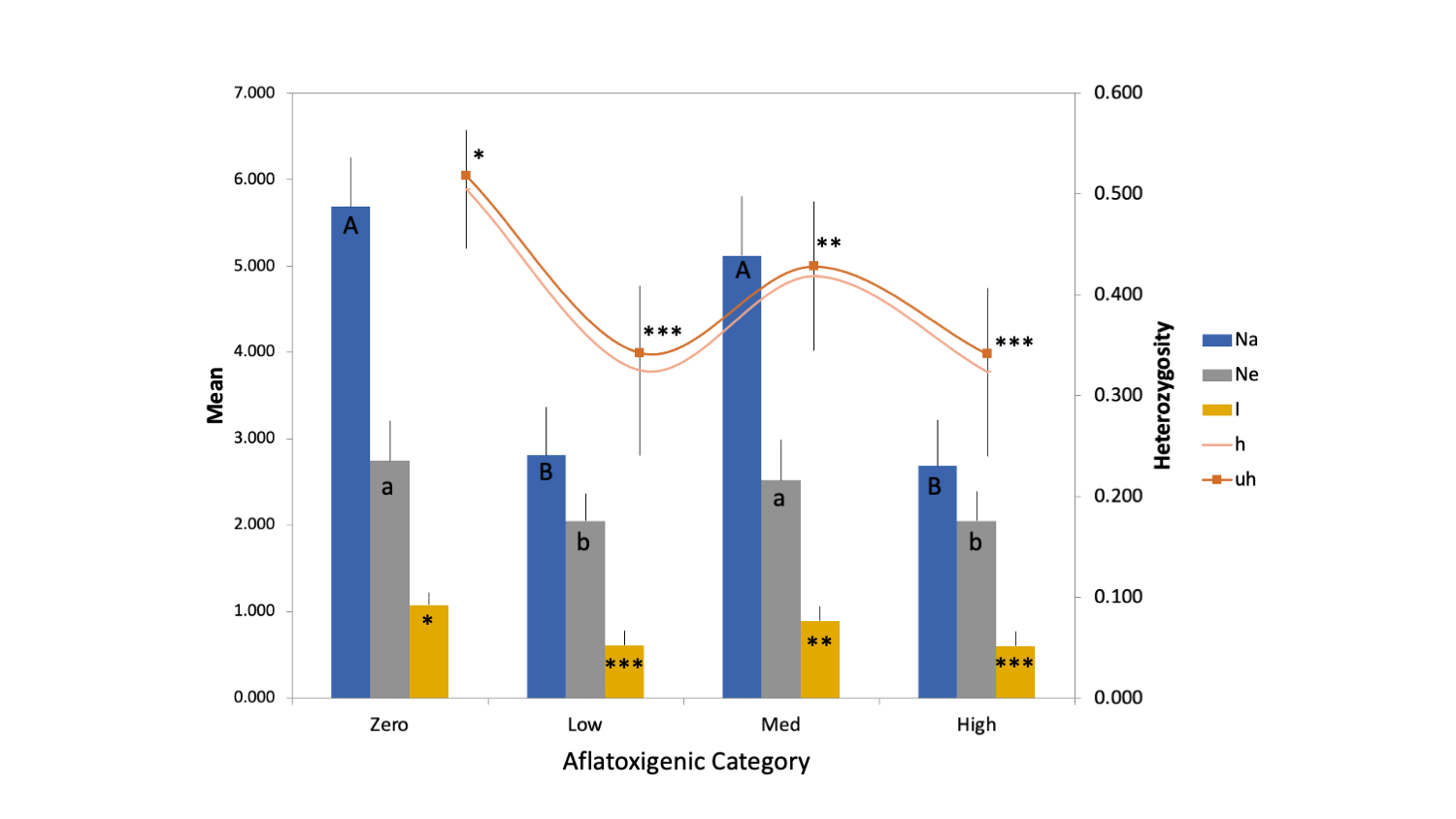

Supplement: Supplementary file 1 [file foods-12-03864-s001.zip › New Supplemental files/Figure S1 Allelic Diversity.tiff]
